# Adult-Born Neurons in the Hippocampus Are Essential for Social Memory Maintenance

**DOI:** 10.1523/ENEURO.0182-20.2020

**Published:** 2020-12-17

**Authors:** Elise C. Cope, Renée C. Waters, Emma J. Diethorn, Kristen A. Pagliai, Carla G. Dias, Mumeko Tsuda, Heather A. Cameron, Elizabeth Gould

**Affiliations:** 1 Princeton Neuroscience Institute and Department of Psychology, Princeton University, Princeton, NJ 08544; 2 National Institute of Mental Health, National Institutes of Health, Bethesda, MD 20892

**Keywords:** adult neurogenesis, dentate gyrus, hippocampus, social dominance, social memory, TK-GFAP

## Abstract

Throughout adulthood, the dentate gyrus continues to produce new granule cells, which integrate into the hippocampal circuitry. New neurons have been linked to several known functions of the hippocampus, including learning and memory, anxiety and stress regulation, and social behavior. We explored whether transgenic reduction of adult-born neurons in mice would impair social memory and the formation of social dominance hierarchies. We used a conditional transgenic mouse strain [thymidine kinase (TK) mice] that selectively reduces adult neurogenesis by treatment with the antiviral drug valganciclovir (VGCV). TK mice treated with VGCV were unable to recognize conspecifics as familiar 24 h after initial exposure. We then explored whether reducing new neurons completely impaired their ability to acquire or retrieve a social memory and found that TK mice treated with VGCV were able to perform at control levels when the time between exposure (acquisition) and reexposure (retrieval) was brief. We next explored whether adult-born neurons are involved in dominance hierarchy formation by analyzing their home cage behavior as well as their performance in the tube test, a social hierarchy test, and did not find any consistent alterations in behavior between control and TK mice treated with VGCV. These data suggest that adult neurogenesis is essential for social memory maintenance, but not for acquisition nor retrieval over a short time frame, with no effect on social dominance hierarchy. Future work is needed to explore whether the influence of new neurons on social memory is mediated through connections with the CA2, an area involved in social recognition.

## Significance Statement

Adult hippocampal neurogenesis has been implicated in behaviors linked to the hippocampus, including social behavior. We used a conditional transgenic mouse line to reduce adult-born neurons and explored social memory and social dominance hierarchy formation. We found that mice with reduced numbers of new neurons were unable to recognize conspecifics as familiar with a long delay after initial exposure but were able to recognize them as familiar with a short delay. We did not observe changes in social dominance as measured by home cage behavior or tube test performance in mice with reduced numbers of new neurons. These data confirm and extend previous reports to show that adult-born neurons are essential for maintenance, but not for acquisition or short-term retrieval of social memories, nor for social dominance.

## Introduction

New granule cells are continuously added to the rodent hippocampus throughout life (Jessberger and Gage, 2014; Kuhn et al., 2018). These new neurons form connections with inhibitory interneurons in the hilus, dentate gyrus, and the CA3 (Restivo et al., 2015; Drew et al., 2016) as well as with excitatory pyramidal cells in the CA3 and CA2 (Toni et al., 2008; Llorens-Martin et al., 2015). Accumulating evidence suggests that the CA2 region is involved in social recognition memory (Hitti and Siegelbaum, 2014; Stevenson and Caldwell, 2014). Studies have shown that lesions and optogenetic silencing of CA2 neurons impair, while optogenetic activation of these neurons improves, social recognition, indicating that these neurons are essential for encoding social information into memories (Hitti and Siegelbaum, 2014; Stevenson and Caldwell, 2014; Smith et al., 2016). Additional studies have shown that the CA2 region plays a role in social aggression (Pagani et al., 2015; Leroy et al., 2018). Since new neurons form connections with CA2 neurons (Llorens-Martin et al., 2015), their elimination may impair social behaviors linked to this region.

Adult neurogenesis has been implicated in many of the known cognitive functions of the hippocampus, including spatial memory, contextual fear memory, and pattern discrimination behavior (for review, see Cope and Gould, 2019). There are also reports suggesting that adult-born neurons are important for social behavior, such as social recognition memory (Monteiro et al., 2014; Garrett et al., 2015; Pereira-Caixeta et al., 2017, 2018) and social dominance (Kozorovitskiy and Gould, 2004; Smagin et al., 2015; Opendak et al., 2016). Using antimitotic drugs and γ irradiation, a report has found that mice with reduced adult neurogenesis have impairments in 24 h social recognition memory (Pereira-Caixeta et al., 2018). Studies using environmental enrichment to enhance adult neurogenesis have found social recognition memory persistence for up to 7 d, and this effect is dependent on adult neurogenesis (Monteiro et al., 2014; Pereira-Caixeta et al., 2017). However, these studies found behavioral effects earlier than the time frame required for new neurons to be functionally incorporated into the hippocampal circuitry and thus be relevant for behavior. Using a conditional doublecortin transgenic line to reduce immature neuron numbers by activation of diphtheria toxin, Garrett et al. (2015) showed that reduced adult neurogenesis impaired social recognition memory when a 2 h interval between novel and familiar mouse exposure was used. We sought to replicate and extend these findings using a different transgenic model of adult neurogenesis reduction and examining whether mice lacking new neurons are completely incapable of social recognition memory even when the interval between exposure to the same conspecific is very short.

Reports have shown that social experiences can have both positive and negative effects on adult neurogenesis, depending on the context. For instance, negative social experiences such as social defeat stress, social isolation, and subordinate social rank position are associated with reduced levels of adult hippocampal neurogenesis (Lu et al., 2003; Kozorovitskiy and Gould, 2004; Lagace et al., 2010; Van Bokhoven et al., 2011; Opendak et al., 2016). Conversely, rewarding social experiences such as sexual experience, dominant social rank position and positive fighting experience are associated with higher levels of adult neurogenesis (Kozorovitskiy and Gould, 2004; Glasper and Gould, 2013; Smagin et al., 2015; Opendak et al., 2016). However, no work has explored whether reduced adult neurogenesis alters agonistic behaviors or dominance hierarchy formation.

We tested the hypothesis that reducing adult neurogenesis impairs social memory and social hierarchy formation. We used mice that express herpes simplex virus thymidine kinase (TK) under the control of the GFAP promoter. With treatment of the antiviral drug valganciclovir (VGCV), adult neurogenesis can be inhibited in TK mice. We found that mice with reduced numbers of adult-born neurons had no change in sociability but lacked social recognition when tested 24 h after exposure to a previously encountered conspecific. We then reduced the delay between the first and second exposure from 24 h to 30 min and found that both TK and control mice treated with VGCV exhibited social recognition behavior that was similar to TK and control mice without VGCV. We next explored whether adult neurogenesis is important for social dominance hierarchy by examining home cage behavior and the non-aggressive tube test paradigm. We did not observe any significant changes in social dominance or agonistic behavior after VGCV treatment. Our data suggest that adult-born neurons are required for maintaining, and potentially retrieving, social recognition memories over longer time periods, but not for the acquisition or retrieval of such memories over shorter time frames.

## Materials and Methods

### Animals, experimental design, and VGCV treatment

**Figure 1.**
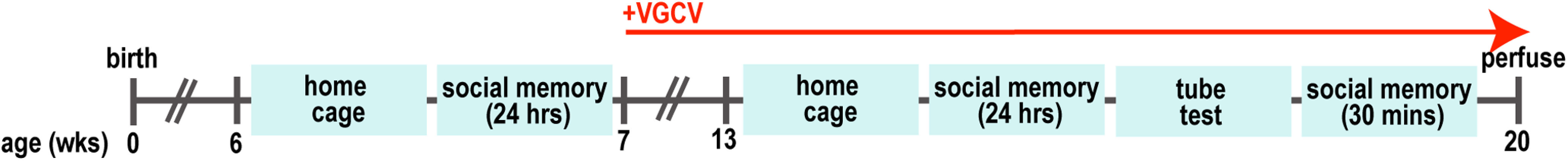
Experimental timeline. Six-week-old CD1 and GFAP-TK mice were examined for home cage behavior and social memory with a 24 h delay in between trials. After behavioral testing, CD1 and GFAP-TK mice were treated with VGCV in powdered chow for six weeks. Mice were then reexamined for home cage behavior and social memory with a 24 h delay in between trials at ∼13 weeks of age. Mice then underwent social hierarchy testing using the tube test at ∼17 weeks of age and then retested for social memory with a 30 min delay in between trials at ∼19 weeks of age. Mice were perfused at the end of the study.

All animal procedures were performed in accordance with the Princeton University animal care committee’s regulations and were in accordance with the guidelines of the National Research Council’s Guide for the Care and Use of Laboratory Animals. Transgenic mice expressing herpes-simplex virus-TK under the GFAP promoter were provided from the Cameron lab. Heterozygous GFAP-TK mice were generated at National Institute of Mental Health by crossing CD1 male mice with heterozygous GFAP-TK female mice. At PND15, mice were genotyped using Transnetyx. Both male and female CD1 and GFAP-TK mice were used for this study (*n* = 24 for each genotype with roughly equal numbers of mice of each sex). All mice were housed four per cage in Optimice cages on a reverse 12/12 h light/dark cycle. For social memory testing, all mice were housed four per cage by sex. For home cage and tube test behavior, all mice were housed four per cage by genotype and sex. Littermates were housed together when possible. Individual animals within a cage were identified by unique brown hair dye markings. Beginning at approximately six to seven weeks of age, home cage behavior and social memory were assessed as described below (Fig. 1). After this, mice of both genotypes were fed VGCV mixed in powdered chow (227-mg VGCV/kg chow) 5 d/week, alternating with standard pellet chow for 2 d. Social memory, home cage behavior, and social hierarchy in the tube test were then retested/tested after six weeks of VGCV treatment. This time frame was chosen because it takes approximately four to six weeks after their production for new neurons to become functionally incorporated into the hippocampal circuitry and contribute to behavior (Denny et al., 2012; Kee et al., 2007). VGCV treatment continued throughout the rest of behavioral testing until perfusion.

### Social memory testing

To assess social memory, the direct social interaction test was conducted in an open-field box (23 × 25 × 25 cm) in low light (10–20 lux) and during the active cycle for mice (dark). Each mouse underwent social memory testing 3 times (once before VGCV treatment and twice after six weeks of VGCV treatment). This test consists of three trials separated by either 24 h or 30 min. Mice were first habituated to the behavior testing room for at least 30 min before testing. Mice were then habituated to the box for 5 min on the initial day of testing before each bout of behavioral testing. In trial 1, the test mouse and a never-before encountered mouse (novel 1) were placed together in the open field box and allowed to interact for 5 min. Sex-matched young adult CD1 mice were used as novel mice. After this interaction period, the test mouse was returned to their home cage for either 24 h or 30 min and then placed back into the open field arena with the mouse previously encountered in trial 1 (trial 2, familiar) and then a new, novel mouse from a different cage than the first novel mouse (trial 3, novel 2). The time the test mouse spent interacting with the encountered mouse was measured from video recordings for each trial. Social interaction was defined as anogenital or nose-to-nose sniffing, following, or allogrooming that was initiated by the test mouse. In some instances, fighting was observed between the test mouse and novel conspecific. If this occurred, the trial was ended and the animal was not included in the behavior analyses. The excluded mice were: one CD1 male mouse in the 24 h delay after VGCV treatment study and three male mice from each genotype from the 30 min delay after VGCV treatment study.

### Home cage behavior testing

Home cage behavior was recorded under red light for 3 h starting at the beginning of the dark phase. Mouse behavior was observed from video recordings of the session. Videos were scanned for occurrences of agonistic behaviors, behaviors that involved physical conflict between animals. These behaviors include but are not limited to chasing, biting, mounting, fighting, freezing, escape behavior, and defensive behavior. All videos were watched in their entirety at 2–30× speed to identify if this behavior was present. For cages where agonistic behavior was observed, behavior was analyzed for 20 min after the first occurrence of an agonistic act of any mouse. The numbers of instances that an animal exhibited offensive behavior and elicited defensive behavior were recorded.

### Social hierarchy tube testing

The tube test was used to measure social hierarchy as previously described (Fan et al., 2019). The tube test consisted of three phases: habituation to a tube, training to pass through a tube, and social hierarchy testing. Mice were habituated to a short (7.6 cm in length) clear tube (2.3 cm in diameter) in their home cage for 3 d before training. Training and testing were completed in low light and during the active cycle for mice (dark). Mice were habituated to the behavior room 30 min before testing. During training, mice were placed at the entrance of a clear tube (30 cm in length and 2.3 cm in diameter) and then trained to pass through it. Each mouse passed through the tube 10 times, five times through each side. To prevent mice from retreating from the tube, a plastic stick was used to guide the mouse forward if necessary. Following 2 d of training, social hierarchy testing began. On each day of testing, mice were first trained to walk through an empty tube two times, once from each side. Mice were then tested against each cage mate in pairs using a randomized round-robin design. Mice were paired such that each mouse encountered every other mouse within that cage only once per day. Two cage mates were placed at opposite ends of the tubes simultaneously and were released once they met in the middle. The trial was completed when one of the opponents retreated out of the tube. Each cage mate pairing had a loser and a winner, with the loser counted as the first animal with all four paws retreated out of the tube and the winner counted as the animal passing forward through the tube. If neither mouse retreated within a 2 min period, mice were retested against each other after a brief rest in their home cage. Testing continued for eight consecutive days, and social rank for each animal was determined based on the number of wins for each day. The tube was cleaned after every mouse with 5% bleach in water followed by 70% ethanol.

### Histology

Mice were anesthetized with Euthasol and then transcardially perfused with 4% paraformaldehyde (PFA) in PBS for immunolabeling with PSA-NCAM to detect immature neurons in the dentate gyrus. In the dentate gyrus, PSA-NCAM appears to label the same population of cells as doublecortin, a known marker of immature neurons (Nacher et al., 2001; Spampanato et al., 2012). Extracted brains were postfixed for 48 h in 4% PFA and then cryoprotected in 30% sucrose for an additional 48 h. Unilateral coronal sections (40 μm) were collected throughout the entire rostrocaudal extent of the hippocampus using a cryostat (Leica CM3050S). Sections were preblocked in PBS containing 0.3% Triton X-100 and 3% normal donkey serum for 1.5 h at room temperature. Sections were then incubated in preblock solution containing antibody rat anti-PSA-NCAM (1:400, BD Pharminogen) for 24 h at 4°C. Washed sections were then incubated for 1.5 h at room temperature in secondary antibodies consisting of goat anti-rat Alexa Fluor 568 (1:250; Invitrogen). Washed sections were counterstained with Hoechst 33342 (1:5000, Invitrogen), mounted onto slides, and coverslipped with Vectashield (Thermo Fisher Scientific). Slides were coded until completion of the data analysis. Cell densities for immature neurons (PSA-NCAM) were analyzed on three neuroanatomically-matched sections of the dorsal dentate gyrus (Franklin and Paxinos, 2008) using a BX-60 Olympus microscope. The number of PSA-NCAM+ cells was determined using a 100× oil objective. The granule cell layer area was outlined at low power (4× objective) using Stereo Investigator software (MBF Bioscience). Cell densities were then determined for each animal by taking the total number of PSA-NCAM+ cells and dividing it by the volume of the granule cell layer (area multiplied by 40 for thickness of cut section). Representative sections were collected using a 20× objective on Leica TCS SP8 confocal.

### Statistical analyses

All behavioral and histologic analyses were performed by an experimenter who was blind to the experimental group. Data from the social memory experiments were first analyzed by three-way ANOVA (sex × genotype × trial). No significant interactions were observed in these analyses (Table 1), so the male and female data were combined and social investigation times were analyzed using a repeated measures two-way ANOVA (genotype × trial) followed by Bonferroni *post hoc* comparisons. Difference scores were analyzed using unpaired or paired Student’s *t* tests where appropriate. Because home cage agonistic behaviors were not normally distributed (as determined by Shapiro–Wilk test) and had unequal variances (as determined by Levene’s test), home cage agonistic behaviors were analyzed using an unpaired Mann–Whitney test or a paired Wilcoxon test where appropriate. For this measure as well, no statistical differences of genotype were observed for either sex (Table 1), so the male and female data were combined. Pearson’s correlation coefficient test was used to analyze the association between home cage agonistic behaviors and performance in the tube test. Cell densities were analyzed by unpaired Student’s *t* test. All datasets are expressed as the mean ± SEM, and statistical significance was set at *p* < 0.05 with 95% confidence. GraphPad Prism 8.2.0 (GraphPad Software) was used for statistical analyses and graph preparations.

**Table 1:** Statistics for female/male comparisons in overall effect of genotype on behavior

Experiment	Statistics	Results
Social investigation pre-VGCV (24 h)	Three-way ANOVA	Sex × genotype × trial: *F*_(2,82)_ = 1.269, *p *=* *0.2865
Social investigation post-VGCV (24 h)	Three-way ANOVA	Sex × genotype × trial: *F*_(2,86)_ = 0.0807, *p *=* *0.9226
Social investigation post-VGCV (30 min)	Three-way ANOVA	Sex × genotype × trial: *F*_(2,74)_ = 0.7855, *p *=* *0.4596
Agonistic behavior difference scores	Mann–Whitney	♀ CD1 vs TK: *U*_(18)_ = 40, *p *=* *0.4947 ♂ CD1 vs TK: *U*_(18)_ = 31.50, *p *=* *0.1089

Statistical analyses demonstrating no overt differences between males and females in the effects of genotype on social behavior measures. Social memory data were analyzed using three-way ANOVA. Agonistic behavior data were analyzed using Mann–Whitney *U* tests. In all experiments, the effects of genotype with or without VGCV were similar in females and males.

## Results

### Mice expressing the GFAP-TK transgene have normal social memory behavior

**Figure 2.**
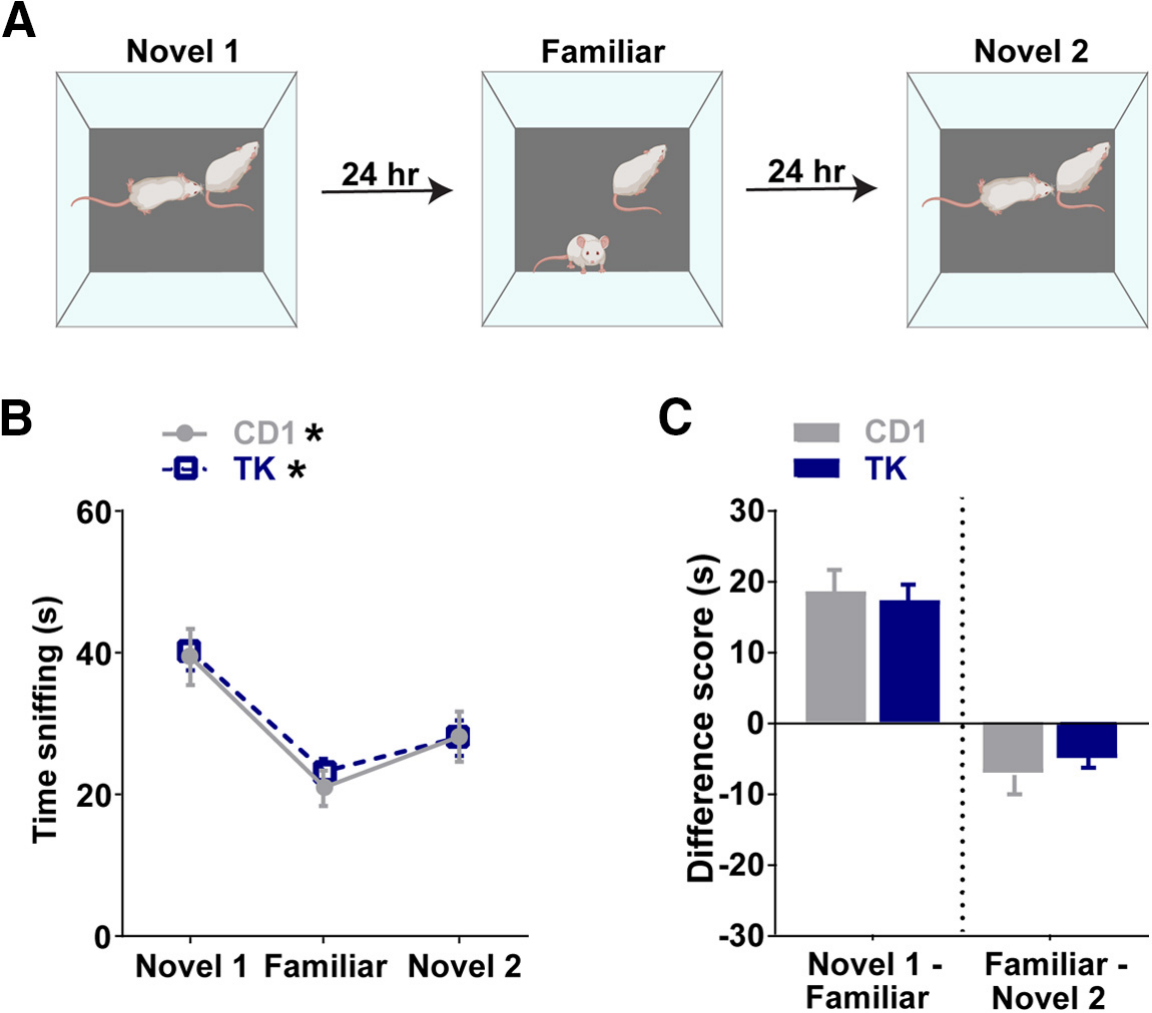
Mice expressing the GFAP-TK (TK) transgene have normal social memory behavior. ***A***, Schematic of three-trial social memory test. ***B***, Both CD1 and TK mice without VGCV treatment decrease their investigation times for a familiar mouse and increase their investigation times for a new novel mouse (novel 2). ***C***, There was no change in difference scores (novel 1 minus familiar or familiar minus novel 2) between genotypes; *n* = 22 for CD1 and *n* = 23 for TK. Bars represent mean ± SEM; *p <* *0.05.

To assess whether the GFAP-TK transgene alters social memory, we first measured social memory using a three-trial paradigm in CD1 and TK mice before receiving VGCV (Fig. 2*A*). Mice were exposed to a novel mouse in trial 1 (novel 1, day 1) and then reexposed to the same mouse in trial 2 (familiar, day 2). In trial 3, the mice were exposed to a new, novel mouse (novel 2, day 3). Social memory was measured by the subject mouse’s reduced sniffing time with the previously encountered mouse in trial 2 and the increased sniffing time with a new novel mouse (novel 2) in trial 3. Repeated measures two-way ANOVA showed that time spent sniffing the stimulus mouse changed over the testing period (two-way ANOVA; effect of trial: *F*_(1.932,83.08)_ = 49.5, *p *<* *0.0001; effect of genotype: *F*_(1,43)_ = 0.07489, *p *=* *0.7857; trial × genotype: *F*_(2,86)_ = 0.2034, *p *=* *0.8164; Fig. 2*B*). *Post hoc* comparisons revealed that CD1 and TK mice had normal social memory such that they decreased their interaction times with a familiar mouse (CD1, *p *<* *0.0001; TK, *p *<* *0.0001) and increased their interaction times with a novel mouse (novel 2), although this did not quite reach significance for CD1 mice (CD1, *p *=* *0.0634; TK, *p *=* *0.0361). There was no difference in time sniffing between genotypes on any trial (novel 1, *p *>* *0.9999; familiar, *p *>* *0.9999; novel 2, *p *>* *0.9999). Furthermore, the sniffing time difference scores were similar between genotypes (novel 1 minus familiar, *t*_(43)_ = 0.3590, *p *=* *0.7213, familiar minus novel 2, *t*_(43)_ = 0.6909, *p = *0.4933; Fig. 2*C*).

### Pharmacogenetic reduction of adult neurogenesis impairs 24 h social memory

**Figure 3.**
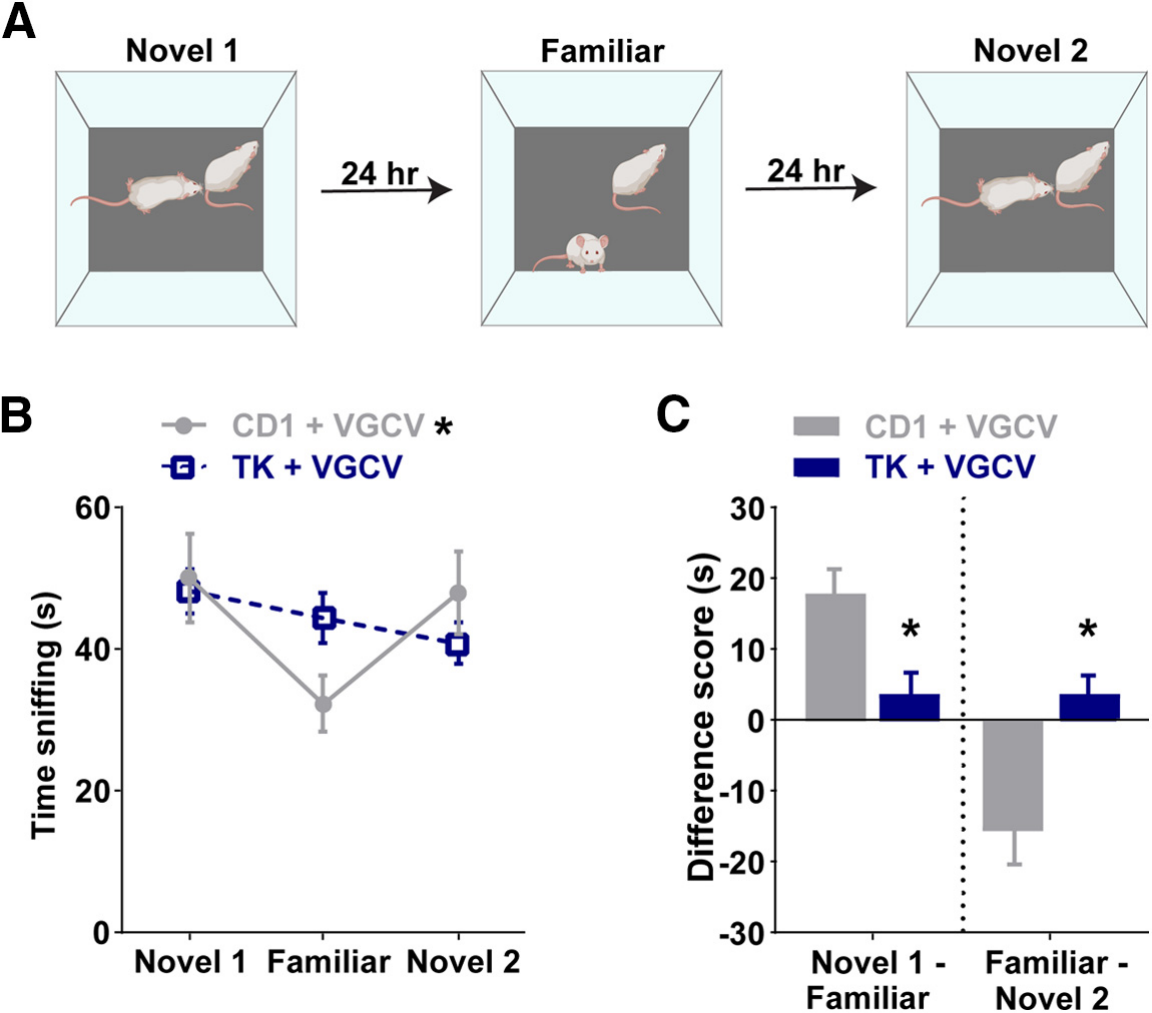
Pharmacogenetic reduction of adult neurogenesis impairs long-term social memory. ***A***, Schematic of three-trial social memory test with a 24 h delay between trials. ***B***, Compared with CD1 mice treated with VGCV, GFAP-TK (TK) mice treated with VGCV do not decrease their investigation times for a familiar mouse and do not increase their investigation times for a new novel mouse (novel 2). ***C***, TK mice treated with VGCV have altered difference scores compared with CD1 mice treated with VGCV; *n* = 23 for CD1 and *n* = 24 for TK. Bars represent mean ± SEM; *p < 0.05.

To investigate whether adult-born neurons are important for social memory, we tested social memory in CD1 and TK mice after six weeks of VGCV treatment using the previously described three-trial behavioral paradigm (Fig. 3*A*). Repeated measures two-way ANOVA of time sniffing showed an effect of trial and an interaction between trial and genotype (effect of trial: *F*_(1.905,85.72)_ = 8.528, *p *= 0.0005, effect of genotype: *F*_(1,45)_ = 0.03717, *p *=* *0.8480; trial × genotype: *F*_(2,90)_ = 7.185, *p *=* *0.0013; Fig. 3*B*). *Post hoc* comparisons revealed that CD1 mice treated with VGCV have normal social memory (novel 1 vs familiar, *p *=* *0.0002; familiar vs novel 2, *p *=* *0.0093), whereas TK mice treated with VGCV had reduced social memory such that they did not decrease their investigation times with a previously encountered mouse (*p *=* *0.6463) nor increase their interaction times with a novel mouse (novel 2; *p *=* *0.5948). Compared with CD1 mice, the sniffing time difference scores were significantly reduced in TK mice (novel 1 minus familiar, *t*_(45)_ = 3.009, *p *=* *0.00429, familiar minus novel 2, *t*_(45)_ = 3.575, *p *=* *0.00085; Fig. 3*C*).

### Pharmacogenetic reduction of adult neurogenesis does not alter short-term social memory acquisition or retrieval

**Figure 4.**
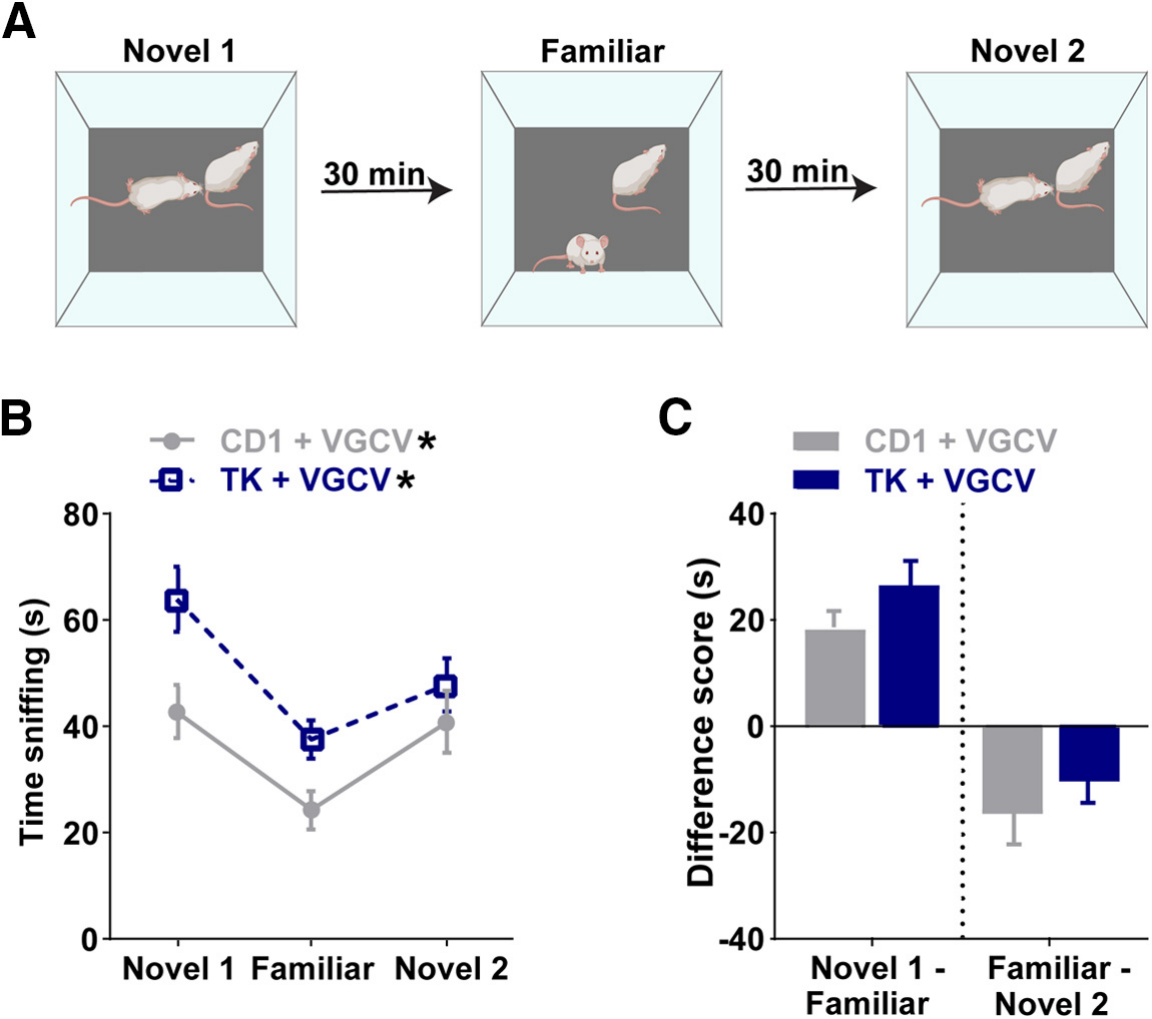
Pharmacogenetic reduction of adult neurogenesis does not alter short-term social memory. ***A***, Schematic of three-trial social memory test with a 30 min delay between trials. ***B***, Both CD1 and GFAP-TK (TK) mice with VGCV treatment decrease their investigation times for a familiar mouse and increase their investigation times for a new novel mouse (novel 2). ***C***, There was no change in difference scores (novel 1 minus familiar or familiar minus novel 2) between genotypes treated with VGCV; *n* = 20 for CD1 and *n* = 21 for TK. Bars represent mean ± SEM; *p < 0.05. For comparison of mice with 24 h delay and 30 min delay paradigms, see Extended Data Figure 4-1.

We explored the possible role of adult-born neurons in social memory (e.g., acquisition, maintenance, or retrieval) by testing CD1 and TK mice treated with VGCV using the three-trial paradigm, but with a shorter delay between trials (30 min; Fig. 4*A*). Repeated measures two-way ANOVA of time sniffing showed an effect of trial and an effect of genotype (effect of trial: *F*_(1.645,64.16)_ = 20.28, *p *<* *0.0001, effect of genotype: *F*_(1,39)_ = 5.925, *p *=* *0.0196; trial × genotype: *F*_(2,78)_ = 2.006, *p *=* *0.1414; Fig. 4*B*). *Post hoc* comparisons revealed that CD1 and TK mice treated with VGCV had normal social memory with a shorter delay between trials such that they decreased their interaction times with a familiar mouse (CD1, *p *<* *0.0001; TK, *p *<* *0.0001) and increased their interaction times with a novel mouse (novel 2), although this did not reach significance for the TK mice (CD1, *p *=* *0.0273; TK, *p *=* *0.0610). It is worth noting, however, that CD1 mice had lower time sniffing on trial 1 (novel 1, *p *=* *0.0293) and trial 2 (familiar, *p *=* *0.0420) compared with TK mice. The difference scores were similar between genotypes in novel 1 minus familiar (*t*_(39)_ = 1.4166, *p *=* *0.1645) and familiar minus novel 2 (*t*_(39)_ = 0.9011, *p *=* *0.3730; Fig. 4*C*).

We then compared both groups’ performance on the 30 min delay paradigm to their performance on the 24 h delay paradigm. The sniffing time difference scores of CD1 mice with a 30 min delay compared with a 24 h delay were similar between groups (novel 1 minus familiar, *t*_(38)_ = 0.129, *p *=* *0.898, familiar minus novel 2, *t*_(38)_ = 0.761, *p *=* *0.451; Extended Data Fig. 4-1*A*). Compared with the 30 min delay paradigm, TK mice treated with VGCV showed an effect in the 24 h delay paradigm. The sniffing time difference scores were significantly altered between the 24 h and 30 min delay paradigms in TK mice (novel 1 minus familiar, *t*_(40)_ = 3.705, *p *=* *0.00639, familiar minus novel 2, *t*_(40)_ = 2.309, *p *=* *0.0262; Extended Data Fig. 4-1*B*).

10.1523/ENEURO.0182-20.2020.f4-1 Extended Data Figure 4-1 TK mice have impairments in social memory with a 24-h delay, but not with a 30-min delay between testing phases. ***A***, There was no change in difference scores (novel 1 minus familiar or familiar minus novel 2) between CD1 mice treated with VGCV when the delay is 24 h or 30 min in between testing trials; *n* = 23 for CD1 + VGCV (24 h) and *n* = 20 CD1 + VGCV (30 min). ***B***, VGCV-treated TK mice have lower difference scores with 24-h delays compared to VGCV-treated TK mice with 30-min delays; *n* = 24 for TK+VGCV (24 h) and *n* = 21 for TK + VGCV (30 min). Error bars represent SEM; *p < 0.05. Download Figure 4-1, TIF file.

### Pharmacogenetic reduction of adult neurogenesis does not alter agonistic behavior or social hierarchy

**Figure 5.**
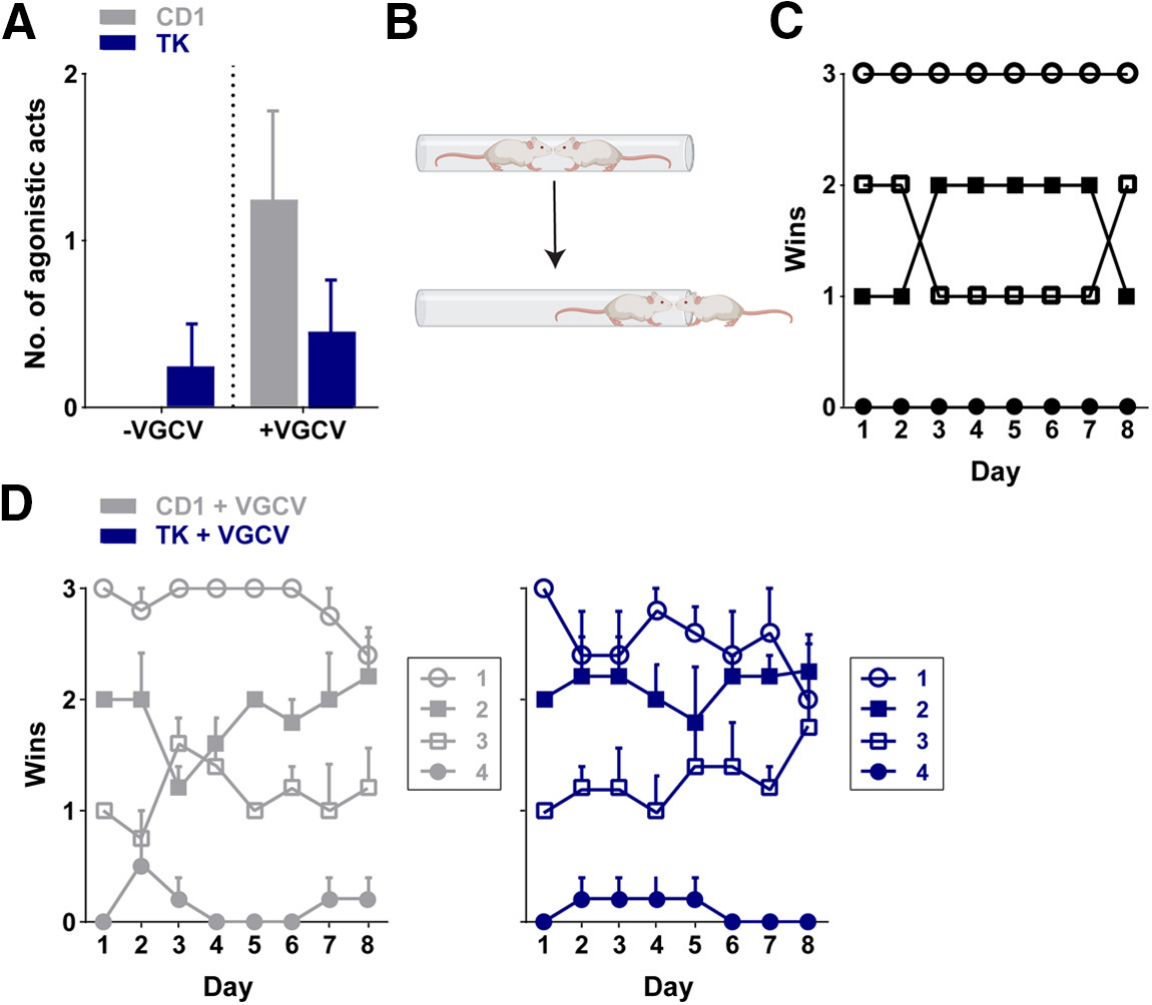
Agonistic home cage behavior and tube-test ranking for social hierarchy in CD1 and GFAP-TK (TK) mice. ***A***, Number of agonistic acts between CD1 and TK mice did not differ before or after VGCV treatment. ***B***, Schematic of the tube test. ***C***, Example of the wins of one cage tested daily over 8 d. Each data point indicates one animal in the cage (*n* = 1 cage with 4 mice). ***D***, Summary graph (*n* = 5 cages/genotype) of the average wins of animals belonging to each rank group from the previous day in CD1 (left) and TK (right) mice. Error bars represent SEM. For individual cage tube test rankings, see Extended Data Figure 5-1.

To assess whether reducing the number of adult-born neurons alters social hierarchy, we examined home cage behavior before and after treatment with VGCV. Before VGCV, there were no agonistic acts (e.g., mounting, chasing, biting, fighting) observed in any of the five CD1 cages and in four of the five TK cages. The number of agonistic acts did not differ between genotypes (*U*_(38)_ = 190, *p *>* *0.9999; Fig. 5*A*). Following six weeks of VGCV treatment, we then measured home cage behavior again and identified agonistic acts in two of the five CD1 cages and two of the five TK cages. The number of agonistic acts did not differ between CD1 or TK mice after VGCV treatment (*U*_(38)_ = 175.5, *p *=* *0.293; Fig. 5*A*), nor was there a significant difference when comparing before or after VGCV treatment in either genotype (CD1: *W*_(38)_ = 15, *p *=* *0.0625; TK: *W*_(38)_ = 6, *p *=* *0.250). The slight increase in agonistic acts between the pre-VGCV and post-VGCV monitoring times in CD1 mice may have been because of the increased age of the mice.

10.1523/ENEURO.0182-20.2020.f5-1 Extended Data Figure 5-1 Tube test ranking for social hierarchy in individual cages of CD1 and TK mice. Wins of each CD1 (***A***) and TK (***B***) cage of mice tested daily over 8 d. Each data point indicates one animal in the cage with four mice per cage. Download Figure 5-1, TIF file.

Because we only observed minimal agonistic acts during the home cage recordings, we were unable to assess social dominance within the home cage. We then explored social hierarchy within a home cage using the non-aggressive tube test paradigm (Fig. 5*B*). We determined an animal’s rank position based on the number of wins of each mouse against its three cage mates over an 8 d period, with the highest rank position being three wins per day (example from a single CD1 cage; Fig. 5*C*). Two of five CD1 cages and three of five TK cages had four consecutive days where animals remained in the same rank position, suggesting that they exhibited stable hierarchies (Extended Data Fig. 5-1). However, in all cages but one TK cage with continued tube test testing, we observed changes in animal’s rank position. We examined the average wins of animals belonging to each rank group from the previous day across testing days and did not find any overt differences between CD1 and TK mice (Fig. 5*D*). We also explored the relationship between agonistic behavior in the home cage and wins in the tube test. Animals with higher levels of agonistic behavior in the home cage did not consistently correspond to a greater number of wins in the tube test. No significant correlation was observed between tube test wins (cumulative wins over the 8 d testing period) and agonistic acts in the home cage after VGCV treatment (CD1 + VGCV: *r *=* *0.1132, *p *=* *0.6347; TK + VGCV: *r *=* *−0.2966, *p *=* *0.2041).

### VGCV reduces the number of adult-born neurons in the dentate gyrus of TK mice

**Figure 6.**
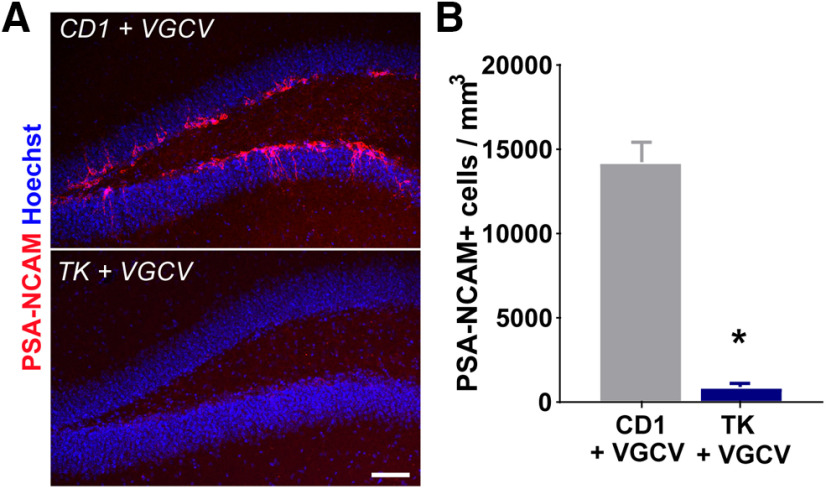
GFAP-TK (TK) mice treated with VGCV have fewer new neurons compared with CD1 mice treated with VGCV. ***A***, PSA-NCAM immunolabeling in the dentate gyrus from CD1 and TK mice treated with VGCV (PSA-NCAM, red; Hoechst, blue). Scale bar = 75 μm. ***B***, The density of PSA-NCAM+ neurons in the dentate gyrus was lower in TK mice treated with VGCV; *n* = 12 per genotype. Error bars represent SEM; *p <* *0.05.

After perfusion, we verified that VGCV was effective in reducing adult neurogenesis in the dentate gyrus of TK mice compared with CD1 mice. Examination of immature neuronal marker PSA-NCAM revealed that TK mice treated with VGCV showed a robust decrease in the density of immature neurons in the dorsal dentate gyrus (*t*_(22)_ = 11.01, *p *<* *0.0001; Fig. 6*A*,*B*). There was no difference in the volume of the dentate gyrus between CD1 or TK mice treated with VGCV (*t*_(22)_ = 2.454, *p *=* *0.808). It should be noted that because the design used in this study was within-subjects, we were not able to quantify immature neurons before VGCV treatment in TK mice. Previous findings suggest that TK mice do not differ in the number of new neurons in the dentate gyrus from their CD1 littermates before VGCV treatment (Snyder et al., 2011; Briones BA, Pisano TJ, Pitcher MN, Haye AE, Diethorn EJ, Engel EA, Cameron HA, Gould E, unpublished observations).

## Discussion

We investigated social memory and social dominance hierarchy in transgenic mice with reduced adult neurogenesis. Unlike mice with normal levels of adult neurogenesis, we found that TK mice treated with VGCV investigated familiar mice as if they were novel 24 h after the initial exposure. By contrast, mice with reduced adult neurogenesis were able to recognize novel conspecifics as familiar when the delay between initial exposure and the second exposure was brief (30 min). This suggests that new neurons are not necessary for the acquisition and retrieval of short-term social memories, but are involved in maintaining, and potentially retrieving, social memories over longer periods of time. We did not observe any changes in mice with reduced adult neurogenesis compared with controls in either agonistic behaviors or social dominance in a home cage or by tube test assessment. Collectively, these findings suggest that adult-born neurons are essential for aspects of social memory that last beyond a 30 min interval.

Some studies suggest that social dominance status influences adult neurogenesis in the dentate gyrus. Studies using a “visible burrow system” in rats, a spatially and socially complex laboratory habitat used to encourage the formation of a dominance hierarchy, have found that subordinate rats have fewer new neurons than social dominants (Kozorovitskiy and Gould, 2004; Opendak et al., 2016), a finding that is consistent with research in mice showing that “positive fighting” experience increases the number of new neurons in the dentate gyrus (Smagin et al., 2015). In a stable hierarchy, the enhancement of adult neurogenesis in dominant rats appears to be driven by increased mating behavior (Kozorovitskiy and Gould, 2004; Opendak et al., 2016). By contrast, adult rats living in an unstable hierarchy exhibit reductions in adult neurogenesis that appear to be driven by stress caused by increased aggression (Opendak et al., 2016). These findings raise the possibility that adult-born neurons are involved in social dominance. Here, in combination with home cage agonistic behavior assessment, we used the tube test, which is a non-aggressive test of social dominance (Fan et al., 2019), to explore the potential relationship between adult neurogenesis and social dominance. We were unable to obtain definitive evidence that new neurons are involved in social aggression or in social dominance hierarchy formation. Despite previous work showing that CD1 mice form stable hierarchies (So et al., 2015), neither CD1 controls nor TK mice exhibited consistent results in the tube test, nor in agonistic acts within the home cage, rendering it difficult to determine social rank status. One explanation for this lack of hierarchy formation could be that mice were housed at weaning with their littermates, which likely minimized fighting throughout the study. More challenging home cage environments (e.g., increased competition for resources) that promote higher levels of agonistic behavior might be necessary to investigate the relationship between home cage behavior and dominance rank. In our study, we examined agonistic behavior in both males and females. Previous studies have shown that male and female mice both form dominance hierarchies but use different strategies to form and maintain them (Williamson et al., 2019). Moreover, while males engage in more overt acts of aggression than females, the overall number of agonistic acts is similar between sexes (van den Berg et al., 2015; Choleris et al., 2018). This claim is consistent with our agonistic behavior findings overall, but future studies should investigate whether TK mice with inhibited adult neurogenesis display alterations in agonistic behavior in response to resident-intruder challenges, which can be designed to stimulate increased aggression in both males (Olivier et al., 1995; Yohn et al., 2019) and females (Haug et al., 1986; Newman et al., 2019).

Our findings on social recognition memory are consistent with previous reports using several methods to block adult neurogenesis, including irradiation (Pereira-Caixeta et al., 2018), antimitotic drugs (treatment with either the DNA-alkylating agent temozolomide or central infusion of the mitotic blocker cytosine arabinoside; Pereira-Caixeta et al., 2018), as well as a tamoxifen-inducible diphtheria toxin doublecortin transgenic mice (Garrett et al., 2015). Our data replicate these findings in a different transgenic mouse of adult neurogenesis reduction, and extend these previous studies by showing that adult neurogenesis is not necessary for the acquisition of social memory, nor for its retrieval after a short time period (30 min), as TK mice treated with VGCV were able to acquire and retrieve social memories using this temporal parameter. However, we found that adult born neurons are important for maintaining social recognition when the interval between exposure to the same mouse was 24 h, suggesting that the impairment results from an inability to maintain, and potentially retrieve, social memories over longer periods of time. There are some reports showing that adult-born neurons are important for spatial memory acquisition and retrieval (Gu et al., 2012; Tronel et al., 2015), yet less is known about adult-born neurons and memory maintenance/consolidation. While the dentate gyrus itself has been shown to be important for social memory retrieval (Leung et al., 2018), the link between this brain region and social memory maintenance has not been explored.

Previous studies suggest that sex differences in social learning exist in rodents (Choleris and Kavaliers, 1999). These differences are not large at baseline (Choleris et al., 2013; Karlsson et al., 2015), however, and mostly involve emerging sex differences in social memory after hormone or drug treatment (Choleris et al., 2013; Matta et al., 2017). As noted above for aggressive behavior, male and female mice seem to use different social strategies, but both sexes exhibit comparable evidence of social recognition memory (Karlsson et al., 2015; van den Berg et al., 2015; Choleris et al., 2018). Consistent with this observation, we detected evidence of social recognition memory in untreated male and female CD1 and TK mice, but no significant interaction when sex × genotype × trial was examined for any of the social memory experiments, demonstrating that males and females both displayed similar social recognition memory within a given experiment. It remains possible, however, that significant sex differences in the effects of genotype on social recognition memory would emerge if females were tested at different stages of the estrous cycle, which was not done in the present study. A previous report suggests that social recognition memory is most robust in females when learning occurs during proestrus (Sánchez-Andrade and Kendrick, 2011). This report raises the possibility that activation of new neurons may be augmented by high levels of estrogen, which is consistent with findings that immature neurons express estrogen receptors (Herrick et al., 2006) and that estradiol treatment enhances the activation of new neurons after exposure to a spatial memory task (McClure et al., 2013). Future studies should determine whether estrogen-induced enhancements in social recognition memory in females (Phan et al., 2012) require new neurons.

One possible interpretation of our data is that the behavioral deficit in mice lacking adult neurogenesis reflects a broader impairment in novelty or familiarity detection. Indeed, some of our results lend support to this possibility in that TK mice with inhibited adult neurogenesis show reduced investigation times between the first novel mouse exposure to the second novel mouse exposure. These findings raise the possibility that TK mice with inhibited adult neurogenesis may habituate to social interactions in general and become less interested in sniffing mice, regardless of their novelty. Considered in the context of the broader literature, however, it seems unlikely that adult neurogenesis is important for novelty detection in a more general sense. In a modified novel object recognition test with a 3 min delay between trials, Denny et al. (2012) found in mice lacking adult neurogenesis increased novelty exploration when a novel object replaced a familiar object over repeated exposures to the familiar object. While we found an increase in social investigation times in TK mice treated with VGCV in the first novel and familiar trial of the 30 min delay paradigm that may support this interpretation, there was no difference in investigation times in the initial novel trial between genotypes in the 24 h delay paradigm. Furthermore, other studies have not found impairments in novel object recognition tests with 24 h delays in between recognition trials in mice with reduced new neurons (Jaholkowski et al., 2009; Goodman et al., 2010), suggesting adult neurogenesis may not be necessary for intact novelty or familiarity detection. Reduced adult neurogenesis has been shown to affect social preference such that rodents without adult neurogenesis spend more time with familiar conspecifics over novel conspecifics (Opendak et al., 2016; Pereira-Caixeta et al., 2018). This suggests that they are able to recognize mice, but prefer familiar conspecifics. However, our data do not support this possibility as there was no difference in social exploration times in the familiar trials compared with the novel trials with the 24 h delay between exposure, suggesting no recognition of familiar conspecifics. Collectively, our results likely indicate a more specific deficit in social recognition memory.

In addition to the hippocampus, GFAP+ radial precursors also reside in the subventricular zone where they migrate along the rostral migratory stream and populate the olfactory bulb (Garcia et al., 2004). Pharmacogenetic ablation of adult-born neurons using VGCV in TK mice reduces olfactory bulb neurogenesis (Singer et al., 2009). Although olfactory discrimination is the main function of the olfactory bulb (Abraham et al., 2010), mice lacking adult neurogenesis in this region have no differences in simple odor discrimination tasks (Imayoshi et al., 2008; Sakamoto et al., 2011; Li et al., 2018). Importantly, in our study, we did not see differences in social investigation times between genotypes during the initial novel trial of the longer delay paradigms and we found intact social memory with a shorter delay in between trials. These findings strongly suggest that deficits in olfactory function are not responsible for the inability of mice lacking adult neurogenesis to exhibit evidence of social discrimination in the 24 h paradigm. Additional studies have provided evidence for the postnatal and adult generation of new neurons in other brain regions associated with social behavior, including the amygdala, hypothalamus, and nucleus accumbens (Kokoeva et al., 2007; Ahmed et al., 2008; Staffend et al., 2014). While postnatal neurogenesis in these areas has been less extensively studied, it remains possible that VCGV treatment of TK mice inhibited neurogenesis in these regions, which may have contributed to the deficit in social memory we observed.

Adult-born neurons form connections with neighboring mature granule cells (Luna et al., 2019), with local inhibitory interneurons in the hilus, dentate gyrus, and CA3 (Restivo et al., 2015; Drew et al., 2016), as well as with pyramidal cells in the CA3 and CA2 (Toni et al., 2008; Llorens-Martin et al., 2015). One report linked the ventral CA3 region to social memory (Chiang et al., 2018), and a growing body of literature suggests an important role for the CA2 region in social memory (Hitti and Siegelbaum, 2014; Stevenson and Caldwell, 2014). Furthermore, the CA2, through its connections with the hypothalamus, has been shown to be involved in social aggression (Leroy et al., 2018). It is likely that alterations in the number of new neurons lead to fewer connections to their target cells in CA2. This, in turn, may alter the proper function of the CA2.

This raises questions about the possible mechanisms by which new neurons exert their effects on CA2-dependent behavior. One possibility is that reducing adult neurogenesis could alter neural activity patterns in the CA2 region. Young granule neurons are known to have enhanced synaptic plasticity (Schmidt-Hieber et al., 2004; Ge et al., 2007; Marín-Burgin et al., 2012) and respond with excitation to the neurotransmitter GABA (Ge et al., 2006). New granule neurons also activate local inhibitory circuits that promote strong feed-forward inhibition onto mature granule cells (Drew et al., 2016) and when activated by specific afferents inhibit mature granule cells directly (Luna et al., 2019). Genetic enhancement of adult neurogenesis leads to an overall decrease in excitability while elimination of adult neurogenesis by irradiation leads to an overall increase in excitability in the dentate gyrus (Ikrar et al., 2013). Adult-born neurons have also been shown to directly impact hippocampal network activity (for review, see Tuncdemir et al., 2019). Ablation of adult neurogenesis by irradiation or by chemotherapeutic drugs reduces neuronal oscillations in the dentate gyrus, particularly in the theta frequency (Nokia et al., 2012; Park et al., 2015). In addition to reducing theta, adult neurogenesis inhibition has been shown to increase γ frequency bursts and synchronization of neuronal firing to these bursts in the dentate gyrus (Lacefield et al., 2012). Since reduced adult neurogenesis alters dentate gyrus neural activity, this is likely to lead to disrupted neural activity in the CA2 target region. Previous work has shown that CA2 neurons participate in generating γ oscillations and sharp-wave ripples in the hippocampus (Alexander et al., 2018). Since these neural activity patterns are associated with cognition and social memory (Buzsáki, 2015; Alexander et al., 2017), future work should explore whether reducing adult neurogenesis leads to disrupted neuronal oscillations in the CA2.

## Synthesis

Reviewing Editor: Margaret McCarthy, Univ of Maryland School of Medicine Decisions are customarily a result of the Reviewing Editor and the peer reviewers coming together and discussing their recommendations until a consensus is reached. When revisions are invited, a fact-based synthesis statement explaining their decision and outlining what is needed to prepare a revision will be listed below. The following reviewer(s) agreed to reveal their identity: Liisa Galea, Grigori Enikolopov.

The manuscript addresses the potential role of adult hippocampal neurogenesis in social memory and social hierarchy. The authors suppress neurogenesis in TK-expressing mice using valganciclovir treatment for 13 weeks, expose the mice to a series of behavioral experiments, and assess the number of new neurons.

They find that 7 weeks after the beginning of VGCV treatment mice show pronounced deficit in the social recognition memory test if the encounters were separated by 24 hours. However, when later tested for the social recognition memory in a similar test but with the encounters separated by 30 min, treated mice do not show such deficits. The authors also find that prolonged treatment with VGCV does not alter agonistic home cage behavior and the outcome of the tube test for social dominance. They further show that, as expected, prolonged treatment with VGCV leads to a sharp difference with control CD1 mice in the number of new neurons in the dentate gyrus.

The topic is important and the results are interesting. There are several concerns and comments:

ADOLESCENCE VS ADULT NEUROGENSIS AND BEHAVIOR:

1. The authors chose to give VGCV to the mice starting at week 7. However, this is still prior to adulthood and during the later stages of adolescence and puberty. Given that VGCV will decrease neurogenesis in any brain area harboring neurogenesis during puberty and there are numerous areas that undergo neurogenesis during this time including areas that influence social behavior and memory such as the amygdala, nucleus accumbens, and hypothalamus (Nat Neurosci. 2008;11(9):995-7. doi: 10.1038/nn.2178, PMID: 24339170). How can the authors be sure it is the decrease in the DG that is causing these differences and not the reduction of cells in these other areas also known to be involved in social memory? As it stands it does not seem the authors can claim 1) that it is adult neurogenesis and 2) that it is HPC neurogenesis that is exclusively affected to drive these changes.

2. It is not clear why juvenile social memory is compared to social memory in adulthood. For example on page 4 line 101 - the first social memory tests were done at 6-7 weeks...there is a lot of work indicating that adolescent rodents have different social behavior (and memory) than adult rodents and as such this is not the correct comparison ( I would suggest removing this comparison). Indeed, one can see the age difference in social memory by examining the data from Figure 2B (juvenile) and 3B (adult). It is also notable that fighting was only seen in the adult mice (after the VGCV -indicating differences in sociability in adulthood compared to juvenile mice).

3. In addition, there is a clear difference in agonistic behavior between adult and juvenile periods, as one would expect seen in figure 5A. here the males and females need to be shown and males and females do show differences in agonistic behaviors (as shown by E Choleris’ lab) and these need to be accounted for especially as sexes were combined.

SOCIAL MEMORY AND HIPPOCAMPUS

4. The work of E Choleris is missing from this paper. This lab has done a lot of work on the involvement of the dorsal hippocampus on social memory and should be including, including some sex differences (Matta R, Tiessen AN, Choleris E. Neuropsychopharmacology. 2017 Nov;42(12):2344-2353).

5. Cited references describing the association between social dominance and hippocampal neurogenesis all relate to papers describing rats. Paper by Smagin et al Frontiers Neurosci, 2015 shows that repeated winning increases hippocampal neurogenesis in mice and is thus relevant to the presented study.

6. The mechanisms of short-term memory formation are substantially different from those of long-term memory formation. Therefore, normal short-term memory retrieval in the 30 min delay VGCV-treated TK mice does not rule out the possibility of retrieval impairment (not just maintenance or not only maintenance) in the 24 hr delay group. Experimentally, the task of distinguishing maintenance and retrieval of memory is challenging and requires a sophisticated design of the behavioral experiments.

7. Experimental animals were housed four per cage, in some cases with littermates. These mice may have established a degree of social hierarchy prior to the treatment and testing, even if not as pronounced as would be for fully unrelated mice. This may have affected the outcome of the social dominance tests, including the round-robin design.

DATA AND DATA ANALYSES

8. There are concerns with the statistics. it is not clear why they used a non-parametric Mann-Whitney- these have low power and although they are supposed to be better than parametric tests if the assumptions are not met they are actually a little worse. but they never tell us why they used them in the first place. They also combine males and females but never give us any stats on their statement that “ no overt sex differences were observed” - given they are looking at social behaviors I find this difficult to believe.

9. It is not always clear what the sample sizes are and these should be added to the figure captions.

10. The paper mentions experiments with males and females and states that there were no differences observed and thus the results are presented for both groups combined. It would help to specify, perhaps as Extended data, whether there were no differences in each of the many parameters measured in this study. For instance, given the difference in how social hierarchy is established in male and female mice, it is surprising that both the absolute and the normalized data would be the same for two sexes in both the house cage and tube tests. Both male and female mice were used but on page 8, line 192 the authors suggest there are “no overt sex differences"” It is not clear what is meant by this or how they tested this. But it is very important to show these data as there are numerous sex differences in social behaviors as well as memory.

11. Social memory is regulated by ovarian hormones in female mice. Did the authors investigate estrous cycle? This may have a profound impact on the variables they investigated (Neuropsychopharmacology. 2011 Jul;36(8):1689-702).

12.Pearson’s correlations are not rank - those are Spearman. Which formula was used Pearson’s product moment or Spearman rank?

13. Why did the authors use a Mann-Whitney test? Any violations of homogeneity of variance are better controlled using a separate variance t-test than the Mann-Whitney.

14.Why do the authors think that the TK rats show a decrease in time spent sniffing across the 3 trials in Figure 3 B?

15. Fig. 6 shows the numbers of PSA-NCAM neurons for CD1 and TK mice after 13 weeks of treatment with VGCV. However, the numbers for such mice before the treatment are not presented. The difference between the two strains may not be as pronounced as after the treatment, but knowing those numbers is still important for understanding the extent of suppression of neurogenesis.

16. Why did the authors use PSA-NCAM as a marker of immature neurons instead of DCX? PSA-NCAM is expressed when NCAN NCAM binding is modified and not just on immature neurons.

17. Page 3 line 76 - the VGCV in TK mice reduces but does not eliminate neurogenesis. Please change the wording here

18. The Novel-Familiar and then Familiar vs Novel on the graphs is confusing. Maybe better to call this Novel1 and Novel2?
