# Supplementary figures and images for: Adult-Born Neurons in the Hippocampus Are Essential for Social Memory Maintenance
Source: eNeuro. 2020 Dec 17;7(6):ENEURO.0182-20.2020. doi: 10.1523/ENEURO.0182-20.2020 (PMC7768285; doi:10.1523/ENEURO.0182-20.2020)

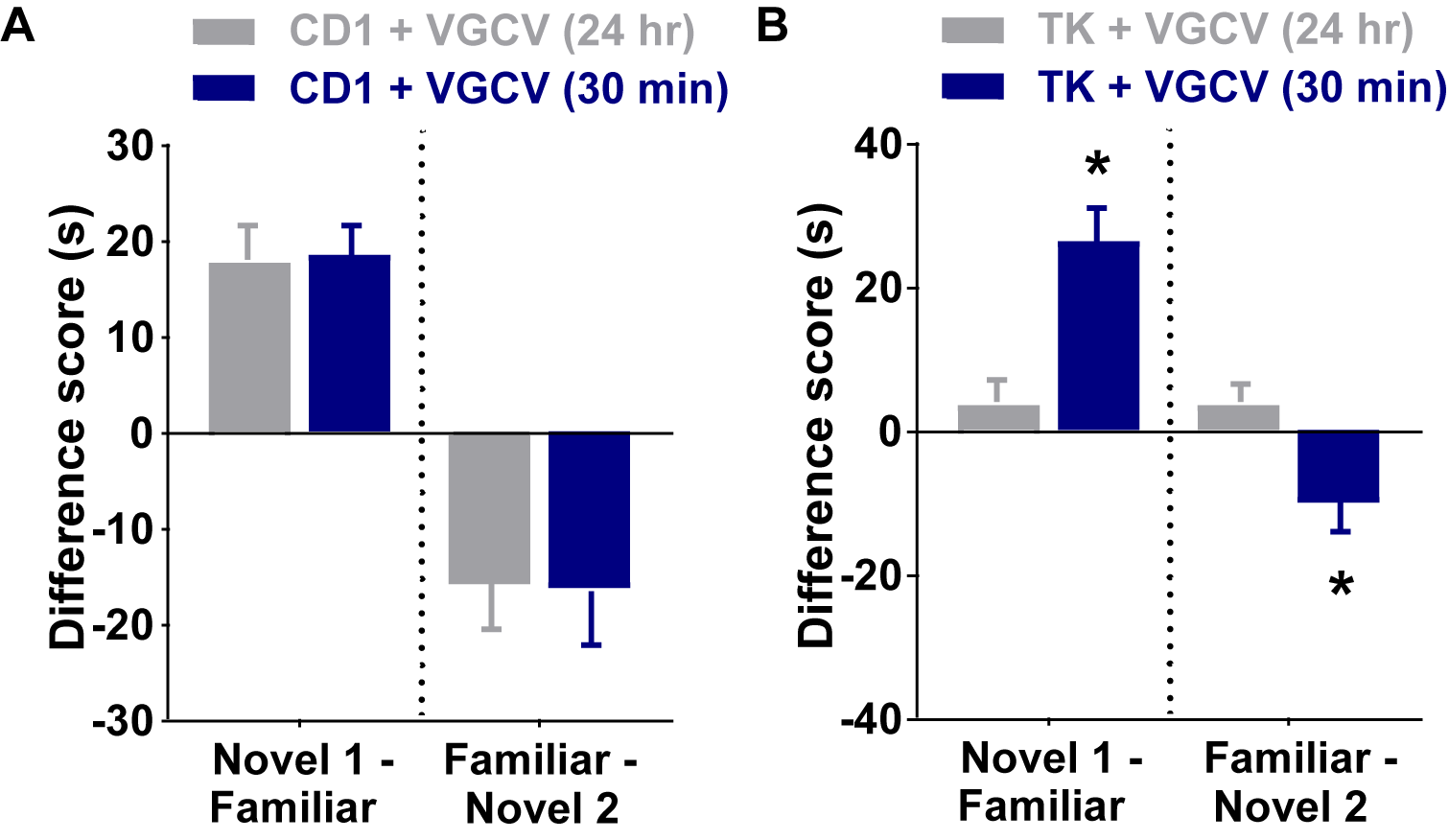

Supplement: Extended Data Figure 4-1 — TK mice have impairments in social memory with a 24-h delay, but not with a 30-min delay between testing phases. A, There was no change in difference scores (novel 1 minus familiar or familiar minus novel 2) between CD1 mice treated with VGCV when the delay is 24 h or 30 min in between testing trials; n = 23 for CD1 + VGCV (24 h) and n = 20 CD1 + VGCV (30 min). B, VGCV-treated TK mice have lower difference scores with 24-h delays compared to VGCV-treated TK mice with 30-min delays; n = 24 for TK+VGCV (24 h) and n = 21 for TK + VGCV (30 min). Error bars represent SEM; *p < 0.05. Download Figure 4-1, TIF file. [file enu-eN-CFN-0182-20-s01.tif]

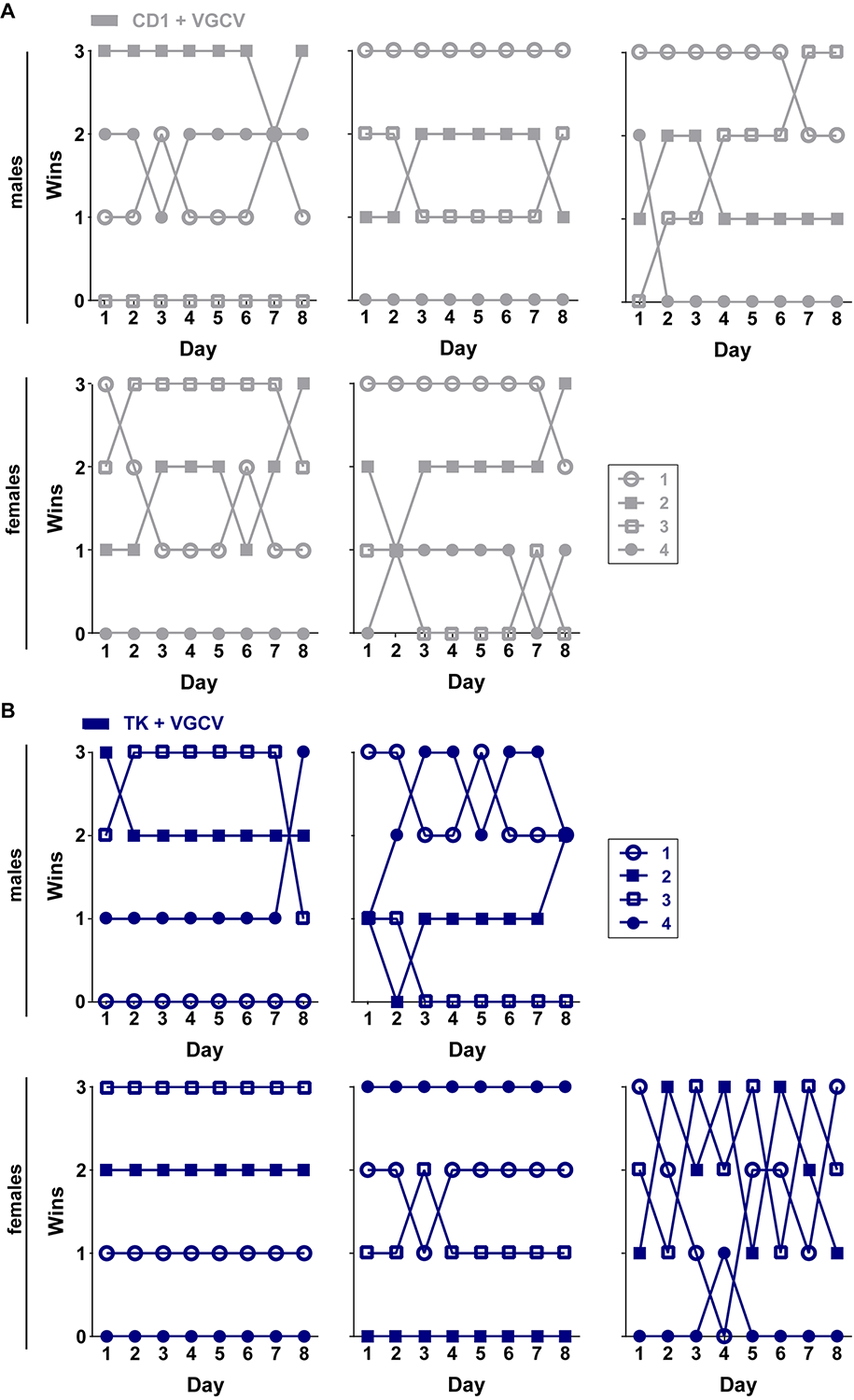

Supplement: Extended Data Figure 5-1 — Tube test ranking for social hierarchy in individual cages of CD1 and TK mice. Wins of each CD1 (A) and TK (B) cage of mice tested daily over 8 d. Each data point indicates one animal in the cage with four mice per cage. Download Figure 5-1, TIF file. [file enu-eN-CFN-0182-20-s02.tif]
